# Health and socio-demographic implications of the Covid-19 second pandemic wave in Israel, compared with the first wave

**DOI:** 10.1186/s12939-021-01445-y

**Published:** 2021-07-02

**Authors:** Daphna Birenbaum-Carmeli, Judith Chassida

**Affiliations:** 1 Faculty of Social Welfare and Health Sciences, University of Haifa, Haifa, Israel; 2 Department of Education, Herzog College, Jerusalem, Israel

**Keywords:** Covid-19, Morbidity rate, Israel, Socioeconomic status, Population density, Elderly population, Vulnerable populations, Minority, Jewish, Arab

## Abstract

**Background:**

Israel’s containment of the first wave of Covid-19 was relatively successful. Soon afterwards, however, in the summer months, a harsher pandemic wave developed, resulting in many more seriously ill and dead Israelis. Israel was the world’s first country to impose a second general lockdown. The present study outlines the early months of Israel’s second pandemic wave, until the imposition of the second general lockdown, and their impact on various communities. The investigation is conducted in conjunction with five sociodemographic variables: population density, socioeconomic status, rate of elderly population, minority status (Jewish / Arab identity) and religiosity (Ultra-Orthodox vs. other Jewish communities).

**Methods:**

The analysis is based on a cross sectional study of morbidity rates, investigated on a residential community basis. Following the descriptive statistics, we move on to present a multivariate analysis to explore associations between the five aforementioned sociodemographic variables and Covid-19 morbidity in Israel in the early second pandemic wave vs. the first Covid-19 outbreak.

**Results:**

Both the descriptive statistics and regressions show morbidity rates to be significantly and positively associated with communities’ population density and significantly and negatively associated with socioeconomic status (SES) and the size of elderly population. These results differ from Wave I morbidity, which was not significantly associated with SES. Another difference vis-a-vis Wave I is the rise of morbidity in Arab communities that led to the disappearance of the previously observed significant negative association of morbidity with minority (Arab) status. Exceptional morbidity was found in Ultra-Orthodox Jewish communities.

**Conclusion:**

The second wave of Covid-19 in Israel has profoundly affected marginalized communities characterized by high residential density, low SES and minority status. Other marginalized and disempowered communities have also been badly hit. While acknowledging the potential contribution of various possible causes, we highlight the policy response of Israel’s government during the early weeks of the second Covid-19 outbreak, suggesting that the severe second wave might possibly be associated with belated, undecided government response during this period.

## Introduction

Israel’s first case of SARS-CoV-2, hereafter referred to as Covid-19, was diagnosed in February 27, 2020. On March 16, with roughly 60 new confirmed cases a day, a general statewide lockdown was declared. The first wave climaxed in early April 2020, with 500–700 new confirmed cases a day and a total of less than 10,000 active cases. Mask wearing in public became obligatory on April 12th, but was officially enforced, though to a limited extent, only a couple of months later. From mid-April, the Covid-19 morbidity started to decline and lockdown restrictions were relaxed. In early June 2020, Israel had roughly 2000 active cases and a death toll of less than 300 persons, representing 33 deaths per million and a fatality rate of 1.67%, substantially lower than in most West European countries and well below the world average of 6.15% at the time.^1^ The pandemic was perceived as effectively contained.

However, in June 2020, the number of new confirmed cases of Covid-19 started to rise, reaching 4000–6000 new cases a day in mid September. The number of deaths approached 1200 or 130 deaths per million. At this point, with a total of roughly 180,000 confirmed cases, 50,000 of which were active, Israel has become the world’s most infected country per population. Whereas severely affected countries like the U.S., India, Spain and France had, at that time, 43, 39, 22 and 10 seriously ill persons per million, respectively, and countries that contained the virus more successfully, like Germany, Sweden and Norway had 3, 1 and 0.2 seriously ill patients per million respectively, Israel had 61. From being one of the most successful countries to have contained the pandemic in its initial outbreak, Israel became the world’s worst affected country. In September 13, 2020, the government applied a second countrywide lockdown. Israel became the world’s first country to impose a second general lockdown.

In this paper, we look into the pandemic burden through a sociodeographic lens: who were the virus new carriers, namely which communities paid the highest toll of the soaring contagion? How was the morbidity in early wave II associated with communities’ sociodemographic characteristics? For the sake of clarity, we refer to the period from the pandemic onset in late February to June 2, 2020 as ‘Wave I’ (WI) and to the subsequent period, from June 3 to the second lockdown in September 13 as ‘Early Wave II’ (EWII). Another terminological note refers to the term ‘morbidity’. As is well known, many people confirmed as Covid-19 carriers are asymptomatic. The use of the term ‘morbidity’ may, therefore, be contested. We acknowledge this ambiguity but, for the sake of reading fluency, continue to use the term ‘morbidity’ as representative of the number of confirmed cases.

Our analysis of the spread of morbidity is conducted on a residential community basis. Within this fraemwork, we investigate the association of morbidity rates with five sociodemographic variables: population density, socioeconomic status (SES), percentage of elderly, minority status (Jewish/Arab descent) and religiosity. As such, this paper is a sequel of our previous analysis of these associations in Israel’s first wave of Covid-19 [1]. The main findings of the previous analysis revealed that in Israel’s Wave I of the pandemic:

- Morbidity rates were significantly and positively associated with population density.
- In the Jewish sector, morbidity rates were significantly and negatively associated with SES and percentage of elderly population.
- In the Arab sector, SES and percentage of elderly population were not significantly associated with morbidity.
- Arab communities, despite their minority status and low SES, sustained significantly lower morbidity than Jewish communities did.

## Materials and methods

This paper is a cross-sectional study, investigating rates of morbidity in Israel on a residential community basis. The analysis of Early Wave II figures was conducted in a similar format to that of Wave I: The dependent variable is Israel’s cumulative Covid-19 morbidity in EWII, spanning from June 3 to September 13, when a second lockdown was declared. Notably, this period does not fully capture the second wave, which lasted well into October, but rather, the weeks and months that led up to the second general lockdown. The numbers of confirmed Covid-19 cases are presented per community and per 1000 local community residents. Though the morbidity figures heavily depend on the scope of testing, the official daily morbidity count served as a foundational indicator of local disease spread. As such, it was consistently presented as formative of government decisions and policy.

The sociodemographic variables we explore are those we have used to analyze WI morbidity: community population density, SES, percentage of elderly population and minority status. In addition, in order to refine the results, we subdivided the Jewish sector, distinguishing ultra-orthodox (UO) communities from other Jewish Israelis. (For detailed rationale of choice of variables, see [1])

## Data source

Data was retrieved from Israel’s Ministry of Health Website, which presents daily cumulative morbidity figures.^2^ In order to calculate EWII morbidity, we reduced the cumulative morbidity at the end of WI from the total number of confirmed cases in September 13, 2020. The difference in the number of communities analyzed in WI vs. EWII represents the difference in the number of communities with confirmed cases in each of the scrutinized period. The study encompasses all Israeli communities with Covid-19 cases diagnosed among their residents, which amounted to 188 communities. The communities were classified by the national/religious identity (Arab/Jewish) of the majority of its residents. Jewish communities were also classified by their level of religiosity. On the whole, 111 communities were classified as (non-UO) Jewish, 69 as Arab and 8 as UO communities.

To supplement this data, we retrieved, for each community, figures from Israel’s Central Bureau of Statistics (CBS), depicting the following variables: community’s population density (number of residents/ community’s area of jurisdiction)^3^; SES on a 1–10 scale^4^; Proportion of local population aged > 65 (number of residents older than 65 / community’s total population)^5^; Minority status (Jewish / Arab; mixed cities were classified by the majority population, except for Jerusalem, which was analyzed in proportion to its Arab/Jewish residents); Religiosity (UO, non-UO.)

## Method

Data is presented in two stages. First, descriptive statistics outline the distribution and average figures of the research variables, with a comparison between the two pandemic waves, so as to depict the difference in crude figures. In the second section, a multivariate analysis is carried out in order to assess the association between each variable and a community’s Covid-19 morbidity and to outline these relationships in Israel’s population in the two pandemic waves. Additionally, the regression data about the Jewish sector is used in order to estimate the *‘marginal effect’* of each of the study’s variables on local morbidity rates according to the localities’ level of religiosity.

### Statistical analysis

The statistical analysis is based on the number of aggregate confirmed cases as of September 13, 2020, minus those published on June 2, 2020 for every community that had confirmed cases in it. The statistical analysis was performed by Stata software and reflects the differences and morbidity prediction for this time interval.

## Results

**Fig. 1:**
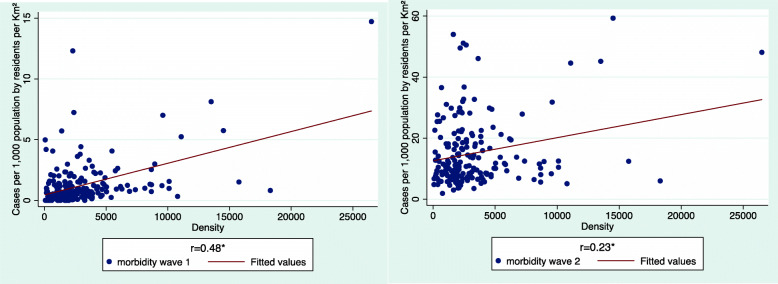
Morbidity rates by community population density, Wave I vs. Wave II

We start the analysis by comparing the distribution of morbidity rates in each scrutinized period in Israel by community population density (Fig. 1). Each figure depicts total accumulated morbidity per 1000 persons in each inspected time interval. It is important to note that though we kept the length of the Y-axis in both figures identical (due to graphic editing considerations), the Y-axis values changed dramatically from 0 to 15 confirmed cases per 1000 in WI, to 0–60 in EWII.

As shown, in both time intervals, morbidity rates were significantly and positively correlated with population density, though the correlation somewhat weakened in EWII (0.48 vs. 0.23 respectively). This decrease represents the spread of morbidity in EWII also to low density communities, as shown in the figure above. Notably, the low density outliers are invariably either Arab or UO communities.

**Fig. 2:**
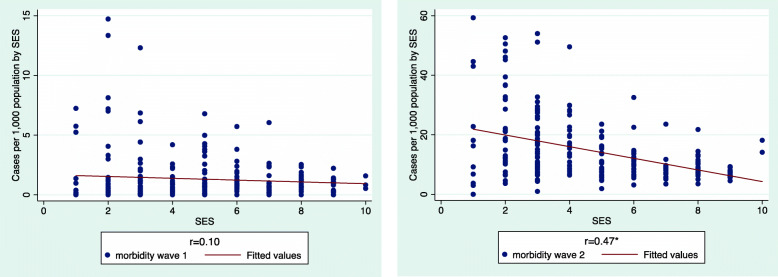
Morbidity Distribution by community SES, Wave I vs. Wave II

Moving on to the correlation with SES (Fig. 2), we note a change from insignificance in WI, to significant negative medium power correlation in EWII, suggesting that in EWII, wealthier communities managed to better protect themselves against the virus while poorer ones became more vulnerable.

The correlation thus shifted from negative but insignificant in WI (r = − 0.10) to negative and significant in EWII (r = − 0.47, *p* < 0.05(. The communities with the highest rates of morbidity (40–60 confirmed cases per 1000), were all clustered at the four lowest SES levels (1–4). Five were OU communities and four were Arab communities, which ranked slightly higher than the OU ones.

**Fig. 3:**
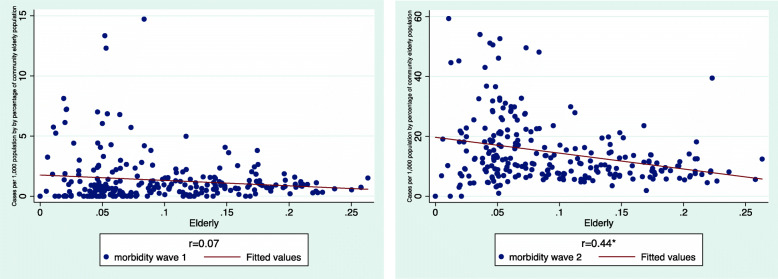
Morbidity by percentage of community elderly population, Wave I vs. Wave II

In terms of the correlation of communities’ percentage of elderly population with morbidity, EWII saw a greatly intensified correlation as compared with WI (Fig. 3).

In WI, the crude data revealed no significant correlation between a community’s percentage of elderly population and its morbidity rates. In EWII, a significant negative correlation was observed, as indicated by the increase of the respective r-values from − 0.07 to − 0.44. These data represent the soaring number of confirmed cases in OU communities, where the percentage of elderly people is extremely low: with a fertility rate of 6.6 children per woman [2], 58% of the UO population are younger than 19 [3]. In this sector, several towns that sustained very high morbidity in WI (*Modiin Ilith, Elad, Rechasim and Bnei Brak*), were among the most infected in EWII as well. An additional contribution to the significant negative association is the rise in morbidity in Arab communities (e.g., *Kafar Bara, Kafar Kasem, Kalanswa and Tira*). Though Arab communities are not as young as OU communities – median ages are 22 and 16 respectively – both these types of communities are younger than the general country population, whose median age is 35 [3, 4]. The percentage of residents aged over 65 in these most affected communities (Fig. 3, EWII), ranged from 2.5 to 8% of the local population.

The correlation of morbidity with minority status has also changed radically. In WI, as noted above, the morbidity in the Arab sector was significantly lower than in Jewish communities [1]. In EWII, morbidity rates in Arab communities were significantly higher than in Jewish communities: 18.50 (*N* = 69 communities) confirmed cases on average per 1000 population vs. 13.05 (*N* = 119) (significant difference: t = 3.37, *p* < 0.05). These figures reaffirm the previously noted extensive contagion in Israel’s Arab communities in EWII.

Given the prominence of UO communities among the worst affected settlements, we conducted a separate comparison, distinguishing UO communities from other Jewish Israeli communities. The difference emerged as formidable. Whereas non-OU Jewish communities had 11.10 confirmed Covid-19 cases per 1000 population on average (*N* = 111), in UO communities (*N* = 8), the respective figure was 40.19. (t = 11.18, *p* < 0.05). Though the number of OU communities is small, the large number of confirmed cases indicates that these communities were significantly more affected than non-UO ones.

Moving beyond descriptive statistics, we probed the association of morbidity rates with each inspected variable and compared the regression models of Israel’s Covid-19 morbidity in the two scrutinized time intervals (Table 1).

**Table 1:** Multilevel Regression Models of morbidity in Israel

Variables	Wave I		Wave II	
	coef. (se)	***β***	coef. (se)	***β***
Density	0.00024* (0.00)	0.439	0.001* (0.00)	0.34
SES	−0.122 (0.07)	−0.150	- 1.09* (0.47)	−0.22
Elderly	−9.16* (2.28)	−0.343	−69.5* (18.2)	−0.37
Minority Status (Jews vs. Arab)	1.682* (0.39)	0.437	0.46 (1.82)	0.02
R^e^	0.381		0.329	
Total (N)	197		188	

*p < 0.05

The regression highlights several differences:

1. Population density has been significantly and positively associated with morbidity rate in both waves.
2. SES was inversely related to morbidity. However, in WI it was insignificant whereas in WEII, it became significant. An increase in one SES level decreased morbidity by 1.09 confirmed cases per 1000 (b = − 1.09, β = − 0.22).
3. The percentage of elderly population remained significantly and negatively associated with morbidity rate, but intensified in EWII.
4. Minority status (i.e., being an Arab community) was significantly and negatively associated with morbidity in WI, with Arab communities sustaining lower probability to morbidity. In EWII, Arab minority status lost its statistical significance, as morbidity in Arab communities rose substantially.
5. The model explains roughly similar portions of the variance in morbidity rate in Israel at the community level: 38% in WI vs. 33% in EWII.

**Fig. 4:**
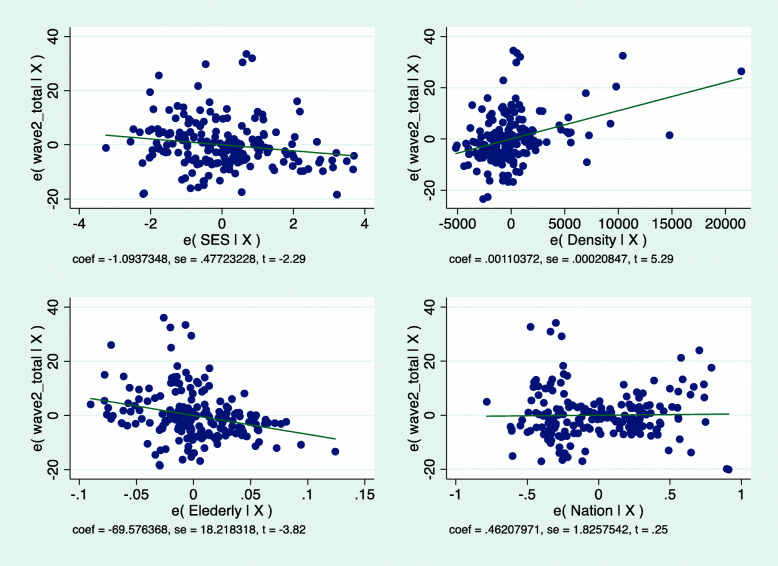
morbidity by population density, SES, percent of elderly population and Minority status

The regression lines presented in Fig. 4 graphically illustrate these EWII trends.

Because the difference in morbidity between UO and non-UO communities emerged as so great in the descriptive statistics, we carried out a Jewish-only regression, distinguishing UO from non-UO Jewish communities (Table 2). As shown, when considering solely the Jewish sector, the three variables – population density, SES and percentage of elderly population – all remain significant. However, UO identity emerges as crucial.

**Table 2:** Multilevel regression model for morbidity rates in UO and non-UO Jewish communities

Variables	Wave I – Jews		Wave 2 - Jews	
	coef. (se)	***β***	coef. (se)	***β***
SES	−0.136 (0.07)	− 0.14	- 1.26* (0.35)	− 0.26
Density	0.000* (0.00)	0.46	0.0005* (0.00)	0.21
Elderly	−9.51* (2.44)	−0.36	−30.7* (14.1)	−0.17
UO vs. Non-UO			17.4* (3.1)	0.43
Total	126		119	
R^2^	0.374		0.619	

*p < 0.05

**Fig. 5:**
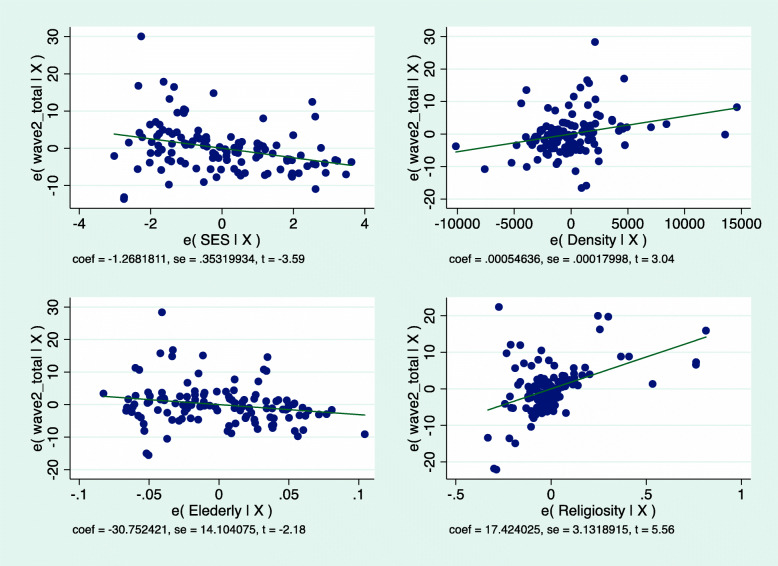
Wave II – Morbidity rates in the Jewish sector by socio-demographic variables and UO religiosity

In the regression, too, OU communities differed significantly from non-UO ones, with 17.4* (3.1) additional confirmed cases per 1000, other variables being equal. Once again, then, morbidity rates in OU communities emerge as much higher than in all others and as the most powerful variable in explaining Covid-19 morbidity in Israel (β = 0.43). The addition of UO religiosity is also significant in terms of R^2^, which explained, after the addition, 62% of the variance in morbidity in the Jewish sector in EWII, as compared to 37% in WI (when UO religiosity was not included in the analysis). Figure 5 graphically depicts the respective regression lines for the Jewish sector.

Upon concluding, we explored the marginal effect of the socio-demographic variables in interaction with UO religiosity in Jewish communities in Israel.

**Fig. 6:**
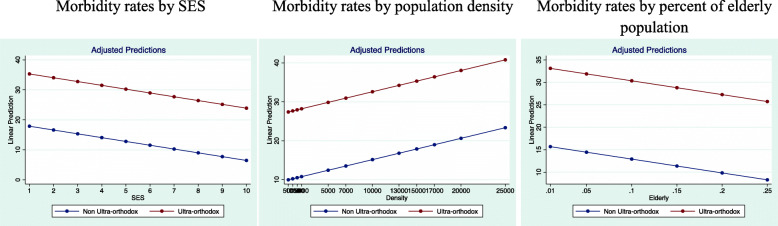
Marginal effect of socio-demographic variables in OU and non-UO Jewish communities in Israel

Figure 6 presents the predicted probabilities of morbidity rate in UO communities vs. other Jewish communities, assuming that the other variables are at average values. As seen, UO communities are likely to sustain a much higher morbidity at every given level of each of the tested variables.

To sum up, the regression models revealed the following changes between WI and EWII:

1. The significant positive association of morbidity with communities’ population density persisted.
2. The negative association of morbidity with community’s SES intensified and became significant in the general country model. (In WI, SES was significant only in the Jewish sector.)
3. The significant negative association of morbidity with the percentage of a community’s elderly population has been maintained and intensified.
4. The negative significant association of morbidity with Arab minority status, which was observed in WI, disappeared in EWII, when the morbidity rates in Arab communities have risen and even surpassed those of Jewish communities, though not to a significant extent.
5. Great gap was observed among UO and other Jewish communities, with the former suffering significantly elevated morbidity rates.

## Discussion

Various reasons can be suggested as underlying the steep rise in morbidity in Israel in EWII. The natural course of the disease; fast release of WI lockdown; lack of government response to the subsequent rise in morbidity; unclear government instructions after WI lockdown; breaches of the regulations by senior public figures, including the state’s President, Prime Minister, Ministers and senior military officers; breaches of the regulations by ‘rank and file’ Israelis due to economic, social and religious pressures; lack of state assistance to people in various forms of need; large public gatherings, mostly for religious rituals, especially during the holidays, attended by both Jewish and Muslim Israelis; ongoing government overlooking of these various breaches.^6^^,^^7^ Notwithstanding these factors, the gap between WI and EWII morbidity was exceptional in Israel. First, the ‘remission’ between the two waves was shorter than in other countries, spanning roughly along the month of May 2020, vs. 3–4 months in Germany, or in badly hit Italy and other European countries.^8^ Second, the increase in the severity in EWII was momentous. Whereas the tenfold rise in confirmed cases can be attributed to intensified testing, the soaring percentage of confirmed cases,^9^ the trebled death toll^10^ and the death rate, which quadrupled from 32 per million in WI to 133 in EWII, all attest to the swift and stark increase in the severity of the pandemic during the summer of 2020.

These figures gain additional weight when compared with those of other countries. To this end, we explored the situation in the period of February to September 2020 in several pandemic stricken European countries of relatively similar population scale: Norway, Austria, Sweden and Ireland. Compared to these countries, in Israel, the gap between the severity of WI and that of EWII was wider than in any of the observed countries (Case Proportion, EWII/ WI was 8.6 in Israel vs. 1.2–2.2 in the other countries), as was the rise in the proportion of death rates (Death Proportion, EWII/ WI was 3.7 in Israel vs. 1.1–1.3 in the other countries). Given Israel’s exceptionally young population, its island-like situation (i.e., its single international airport), its hot summer months that might have slowed down the virus spread, the deterioration is especially alarming, raising questions regarding the factors that might have underlain it.

Several structural factors seem to have obviously facilitated the disease spread. Israel’s population density is extremely high, exceeding those of all OECD country, but the Netherlands and South Korea.^11^ High fertility rates of over three children per woman, result in large families that often live in small apartments, sometimes, in multigenerational families, and are therefore more likely to suffer from domestic contagion. Some sectors lead traditional lives that entail large social gatherings for family and religious events. In such communities, breaches of government pandemic dictates were routine. Indeed, reluctance towards government restrictions has widened as the pandemic progressed and gradually included also diffused public criticism and countrywide demonstrations.^12^

What was the government’s response to these dynamics? As mentioned, the government’s strategy has changed dramatically over the scrutinized period. Starting out from tight lockdown, early on in WI, it moved on to withdrawal of social restrictions and reluctant enforcement in EWII. According to the Oxford Government Response Stringency Index (GRSI), based on 18 indicators,^13^ the response of Israel’s government to WI was swift and stringent, followed by a gradual relaxation of restrictions. Interestingly, the government continued to reduce the stringency of its response throughout the summer of 2020, when the morbidity was steeply resurging, with daily confirmed cases rising from several dozen in early June to over 6000 in mid-September. From mid-August, Israel’s government sustained its exceptionally low level of 34 on the GRSI, while Covid-19 morbidity and mortality were soaring.^14^

Notably, between June 29 and August 17, when the number of daily confirmed cases climbed from 800 to 2071, Israel’s government kept lowering the stringency of its response from 75 to 34. In the subsequent month, from August 16 to September 17, Israel’s government retained its response at a stringency level of 34, lower than that of Sweden, while the number of newly diagnosed cases trebled from a 7-day average of 1383 to 4247 and the number of severely ill Israelis climbed by roughly 50%, from 382 to 573.

Under these epidemiological and political circumstances, how did the pandemic spread impact various local subpopulations? As revealed by the preceding analysis, dense, poor, young communities were especially infected. Prominent in this category were UO communities, followed by Arab ones. In UO communities, both the density and poverty have been largely shaped by the communities’ own preference of exceptional fertility rates and low male participation in the labor market, due to prioritization of Judaic studies over gainful employment. In the Arab sector, the crowdedness and poverty were primarily imposed from the outside by decades of discriminatory state policies that marginalized Arab Israelis in education and the labor market. During the pandemic, Arab communities, that were less intensively supported by state allowances and foreign charities (compared to UO communities), were economically injured more pervasively by the lockdowns (Reznikovski, unpublished).

Throughout the pandemic, these two sectors were repeatedly condemned for conducting large scale social and religious gatherings. Several points need to be noted in this context. First, Covid campaigns in Arab^15^ and UO^16^ communities were substantially deficient in the pandemic early days, as compared to campaigns conducted in other communities, resulting in belated and more partial response to the pandemic outbreak. Second, the majority of the communities in both sectors abided by the regulations and significant breaching was observed only in few particular communities. In this respect, the public discourse, which often lumped together ‘the UO’ or ‘the Arabs’ as monolithic entities, was did not do justice to these communities of humble means.

More generally, both the UO and the Arab sectors have been socially and politically marginalized in Israel’s public arena for years. The communities’ members live in their own segregated towns, mostly working in sectorial work places and marrying internally. Even linguistically, in the Arab sector and in substantial portions of the UO sector, the spoken languages – Arabic and Yiddish, respectively – are not the country’s official language (which is Hebrew). Politically, too, despite their dramatically different positions in the state’s power structure, both these sectors have been alienated from Israel’s hegemonic Zionist ethos, from the country’s symbolic center and its national narrative and are have been represented by their own sectoral parties since the founding of the State of Israel. Materially, this alienation manifests also in exclusion from the state’s security bodies. Whereas one can dispute the merits of serving in these bodies, participation in any of them is a source of ample material and social benefits, of which both these sectors are excluded. This separateness has probably contributed to the communities’ approach that underlay pervasive non-adherence to government guidelines during EWII.

What were the internal circumstances of the pandemic in these sectors? In UO communities, numerous families regularly depend on various types of allowances, most of which have remained intact. These allowances, alongside the highly organized community charities that operate regularly in the UO sector, have protected many UO families. However, the closure of the educational system has brought home many teenage children, who normally attend boarding schools, and rendered the small apartments even denser than they routinely are. The congestion of large families in small spaces increased the risk of contagion. In some cases, it also flared up domestic violence. Though family problems are extremely under-reported in UO communities, preliminary accounts estimated that UO domestic violence calls have quadrupled during the pandemic.^17^ The lack of internet access, due to ideological rejection, impacted UO children, who were cut off from their peers and teachers even more than their non-UO peers. Telephone based distant studies were established but worked rather poorly.

In the Arab sector, families and communities sustained over 30% of unemployment and unpaid leave from the early days of the Covid-19 outbreak in Israel. Following this financial decline, 42% of Arab families sunk below the poverty line. Loan applications were, however, mostly declined by banks and other official lenders, thereby forcing people in need to withdraw money from pension savings or else issue grey market loans. Soon thereafter, people who were unable to repay their loans and the high interests they incurred, faced lenders’ violent reactions, which were part of a broader, steep rise in rates of local violent crime. In line with this state of affairs, 30% of Israel’s Arab citizens – twice the percent of Jewish Israelis – have reported, during the pandemic, feelings of insecurity, stress, anxiety and depression and described their psychological state as negative.^18^ Children in Arab families, who had insufficient internet access, could not fully participate in online schooling. Domestic violence has also soared. In the first 7 months of 2020, 50 Arab Israelis were killed due to violence. Eight of the victims were women. Insufficient police presence and distrust in its service greatly contributed to the deterioration.^19^

Problems and strain of various types abounded, however, also beyond these two sectors. The novelty and volunteering that dominated WI gave way to growing routinization, spreading social, psychological and economic distress, especially in disempowered settings. Economic difficulties and concerns also deepened. In order to assess the scope of the impact, we need to look at the Israeli landscape before the pandemic outbreak.

Israel’s cost of living has been extremely high for years, ranked seventh in the world.^20^ The mounting prices affected even the most basic products like food and electricity and contributed to growing inequality and class polarization.^21^ Of all Israeli workers, just over one quarter (27%) are unionized [5, 6], leaving the rest largely unprotected. Small business owners and employees are especially exposed to market fluctuations and have been defenseless in the face of the pandemic-induced commercial slowdown. The first wave broke out when less than 200,000 Israelis were unemployed. The second wave evolved with nearly 900,000 people on unpaid leave or unemployment benefits. The economic impact was colossal.

During the pandemic first months, state support to businesses was minimal and applied only to particular categories.^22^ Bureaucratic hurdles made it hard for beneficiaries to receive these allowances even when they were entitled to.^23^ For many small-scale entrepreneurs, who had taken loans in order to survive the first lockdown and subsequent business decline, the scarce state support and then the second lockdown, were fatal. Numerous small businesses were forced to close down, at first, temporarily and then permanently [7]. A senior economist estimated that some 80,000 businesses, representing a fifth of the country’s businesses, were facing financial collapse, and predicted that hundreds of thousands of workers on unpaid leave, will not manage to go back to work.^24^ The scope of the expected economic crisis has been so momentous that the police has developed plans for tackling the potential tide of crime. As part of this forecast, a steep rise was expected also in the number of new prisoners as well as women who would be forced to turn to sex work.^25^ At that very time, a policy modification allowed company owners to distribute exceptional benefits they had been ‘caging in’ for years, at greatly reduced taxes.^26^

Social crises also proliferated. Women, in general, have been overrepresented among the pandemic various victims. In September 2020, women comprised 62.7% of unemployed Israelis or those on unpaid leave, roughly twice as men.^27^ In the private sphere, women in abusive relationships were under greater threat. During the month of September, domestic violence reports trebled as compared to the previous year.^28^ In specific time intervals, the proportion increased five^29^- to tenfold.^30^ Between March and mid-October 2020, 17 women were killed, most of them, by their partners.^31^ This figure represents a 50% annual increase vis-à-vis the previous decade’s average.^32^

Adolescents and young people have also been vulnerable to the tide of morbidity. The number of calls to a domestic violence hotline for youths aged 18–20 increased by 500% vs. 2019.^33^ Tens of thousands of calls from pupils reported psychological distress to Israel’s Ministry of Education hotline. An official report assessed that roughly a quarter of Israeli school students experienced stress, loneliness, anxiety, domestic violence and risk behaviors in EWII.^34^ With two thirds of the children reporting inability to participate effectively in online learning, damage was also accumulating in the educational sphere. A similar proportion of school pupils said they were spending most of the time on their own.^35^ Children with special needs have been at an even higher risk for various forms of deterioration and harm.^36^

As elsewhere, elderly people were especially hit by the pandemic also in Israel. As of October, the average age of the dead was 79.5 and the median age was 82. Female victims were slightly older (81.9) than the male (77.6).^37^ As common in Israel, many of the people in this age group were accustomed to routine frequent gatherings with family and friends. The pandemic has brought an end to these gathering and exacerbated anxiety and loneliness among many elderly Israelis. In a recent report, half the participating elderly people said they were feeling lonely.^38^ The number of lone elderly people who have died in the pandemic months have risen by 47% as compared to the previous year (ibid).

People who were subjected to social exclusion before the pandemic were particularly harmed by its spread. Prisoners had their home and prison visits cancelled and suffered restricted access to healthcare. Given that roughly 40% of prisoners are diagnosed with chronic illnesses and 73% have had previous psychiatric referrals, and given the growing portion of elderly prisoners, the health implications of the limitations on care services were especially grave in this population.^39^ People living in Israel as undocumented or stateless residents, e.g., foreign workers, asylum seekers or Palestinians and tourists who have overstayed their visas, remained completely vulnerable. Many of these people have lost their jobs and were not entitled to any financial or logistic support from the state. Among asylum seekers, about 80% have become unemployed. Requests for food, especially babies’ food, have doubled, primarily on the part of single mothers.^40^ Due to the fear of contagion, the state has decided to provide pandemic-related medical treatment to these individuals and communities, who are normally prevented from obtaining medical insurance. This support was, however, minimal and in many cases, ineffective as it had not been planned to match the needs of the population at hand.^41^ In a recent letter to the heads of state, community activists described how they had managed to survive the first pandemic wave, with the aid of volunteers and NGOs. However, in EWII, “the economic and psychological situation of our community is catastrophic. The community is in a state of complete uncertainty and helplessness.”^42^

A pandemic is, evidently, a major social, economic and political crisis. As such, it is reflective and formative of its social context. In Israel, years of underfunding of the health and education systems, growing social fragmentation and economic polarization have preceded the pandemic. Already before the pandemic, 18% of Israeli families and 30% of Israeli children lived beneath the poverty line. Among Arab Israelis, 45% of families were below the poverty line. Poverty among women is 21% higher than among men. Gini coefficient index is roughly 10% higher in Israel than in developed countries.^43^ These gaps have all surfaced and intensified when the state has withdrawn from disease containment following WI lockdown and allowed the pandemic to further spread.

Evidently, the prolonged duration of the pandemic has in itself had its impact. Some of the difficulties depicted above piled up in many communities, well beyond Israel. And yet, the striking differences between the government’s policy in the pandemic’s first wave and the early second wave suggest that grater government involvement and support, as well as stricter enforcement might better protect vulnerable populations, including minority, poor and elderly women and men. The very emergence of the second wave and its exceptional enormity, may possibly be related to the belated, undecided government response to the soaring morbidity that placed Israel far below other island countries that managed to contain the pandemic.

Israelis were this badly hit by the covid-19 pandemic. At the time of this writing, nearly 5500 Israelis have died of the disease, representing 600 deaths per million. People suffering all forms of disadvantage have been especially implicated, paying the price of what appeared to be a politicized, reluctant government policy that let the pandemic take its natural, lethal course.

## Limitations

The study’s periodization, namely the definition of each of the scrutinized periods, has some limitations. The first time interval, February – June 2, 2020, which was analyzed in the previous article [1], includes the May remission period. The second scrutinized interval, June 3–September 13, 2020, includes the early phase of the second wave but not the entire pandemic bout. As such, the comparison is not between two ‘full’ pandemic waves, but rather between the whole initial outbreak and the first weeks of the second one.

Another limitation refers to the analysis of Israel’s mixed cities (e.g., Haifa, Acre, Ramle). These communities were classified according to the local majority, i.e., a city with 80% Jewish population was classified as Jewish. In addition to the smoothing of the statistical analysis, this decision was also guided by the (ungrounded) hypothesis that the minority population that lives in a mixed city might possibly adopt some distinct practices that are context dependent. If this is indeed the case, which we cannot substantiate in the present study, then these particular Arab communities may comprise a somewhat distinct subpopulation. We therefore decided to sustain a majority-led classification. One exception to this rule is the city of Jerusalem, where the Arab residents are Palestinians from the occupied West Bank and comprise over a third of the city’s inhabitants. Due to these distinct characteristics, and because it is Israel’s largest city, therefore applying to a larger subpopulation, we divided Jerusalem’s residents according to their Jewish/Arab identities.

## Conclusion

The second wave of Covid-19 in Israel has profoundly affected marginalized communities characterized by high residential density, low SES and minority status. Other marginalized and disempowered communities have also been badly hit. While acknowledging the potential contribution of various possible causes, we highlight the policy response of Israel’s government during the early weeks of the second Covid-19 outbreak, suggesting that the severe second wave might possibly be associated with belated, undecided government response during this period.

## Data Availability

Sources of data are specified in the manuscript.

## Footnotes

1 https://ourworldindata.org/mortality-risk-covid; identical to extrapolations from https://www.worldometers.info/coronavirus/, on May 28, 2020.

2 https://datadashboard.health.gov.il/COVID-19/general

3 https://www.cbs.gov.il/he/pages/default.aspx

4 https://www.cbs.gov.il/he/publications/doclib/1999/local_authorities/pdf/t02.pdf

5 https://data.gov.il/dataset/residents_in_israel_by_communities_and_age_groups/resource/64edd0ee-3d5d-43ce-8562-c336c24dbc1f

6 https://www.maariv.co.il/corona/corona-israel/Article-786852

7 https://www.ha-makom.co.il/post-bnei-brak-walk/ https://www.idi.org.il/articles/32479 https://www.haaretz.co.il/health/corona/.premium-1.9198975

8 https://www.worldometers.info/coronavirus/

9 European CDC – Situation Update Worldwide – Last updated 20 September, Official data collected by Our World in Data

10 https://www.worldometers.info/coronavirus/

11 https://data.worldbank.org/indicator/EN.POP.DNST?end=2018&locations=OE&most_recent_value_desc=true&start=2008 If we omit Israel’s Southern desert, that is almost wholly taken up by military bases and training areas, Israel’s density is substantially higher than that of these countries as well.

12 https://www.themarker.com/allnews/.premium-1.9155401, https://www.ynet.co.il/article/rJY11QwLBw

13 https://www.bsg.ox.ac.uk/research/research-projects/coronavirus-government-response-tracker. The indictors are: School closing, Workplace closing, Cancel public events, Restrictions on gatherings, Close public transport, Stay at home requirements, Restrictions on internal movement, International travel controls, Income support, Debt/contract relief, Fiscal measures, International support, Public information campaigns, Testing policy, Contact tracing, Emergency investment in healthcare, Investment in vaccines, Facial Coverings.

14 https://ourworldindata.org/policy-responses-covid

15 https://www.phr.org.il/wp-content/uploads/2020/10/CovidReport_Heb_digital-the-real-final-version-compressed.pdf, p. 19.

16 https://m.knesset.gov.il/activity/committees/petitions/news/pages/%D7%94%D7%A1%D7%91%D7%A8%D7%94-%D7%A7%D7%95%D7%A8%D7%95%D7%A0%D7%94-%D7%91%D7%9E%D7%92%D7%96%D7%A8-%D7%94%D7%97%D7%A8%D7%93%D7%99.aspx

17 https://www.ynet.co.il/judaism/article/HyZ11xzzew

18 https://www.globes.co.il/news/article.aspx?did=1001343428

19 https://fs.knesset.gov.il/23/Committees/23_cs_bg_578017.pdf

20 https://www.worlddata.info/cost-of-living.php

21 https://www.haaretz.co.il/opinions/.premium-1.9248482

22 https://news.walla.co.il/item/3373969;https://www.hashikma-rishon.co.il/news/38321

23 https://www.funder.co.il/article/111577

24 https://www.haaretz.co.il/opinions/.premium-1.9248482

25 https://www.haaretz.co.il/news/law/.premium-1.9250135

26 https://www.themarker.com/law/.premium-1.9245216, October 19, 2020

27 https://www.haaretz.co.il/news/local/.premium-1.9244015

28 https://www.haaretz.co.il/news/education/.premium-1.9208324

29 https://www.ynet.co.il/article/BycTg8ivD

30 https://www.haaretz.co.il/news/education/.premium-1.8863213

31 https://www.ynet.co.il/article/BycTg8ivD

32 Police data, October 2020

33 https://www.tipulpsychology.co.il/%D7%91%D7%9C%D7%95%D7%92/domestic-violence-covid-19.html

34 https://brookdale.jdc.org.il/wp-content/uploads/2020/05/Children-and-youth-at-risk-and-corona.pdf

35 https://haruv.org.il/wp-content/uploads/2018/11/%D7%AA%D7%A4%D7%99%D7%A1%D7%95%D7%AA-%D7%95%D7%AA%D7%97%D7%95%D7%A9%D7%95%D7%AA-%D7%A9%D7%9C-%D7%99%D7%9C%D7%93%D7%99%D7%9D-%D7%91%D7%A0%D7%95%D7%92%D7%A2-%D7%9C%D7%95%D7%95%D7%99%D7%A8%D7%95%D7%A1-%D7%94%D7%A7%D7%95%D7%A8%D7%95%D7%A0%D7%94-%D7%95%D7%97%D7%99%D7%99%D7%94%D7%9D-%D7%93%D7%95%D7%97-%D7%9E%D7%97%D7%A7%D7%A8-%D7%9E%D7%9B%D7%95%D7%9F-%D7%97%D7%A8%D7%95%D7%91-%D7%90%D7%A4%D7%A8%D7%99%D7%9C-2020-1.pdf

36 https://brookdale.jdc.org.il/wp-content/uploads/2020/05/Children-and-youth-at-risk-and-corona.pdf

37 https://www.haaretz.co.il/health/corona/.premium-MAGAZINE-1.8730699

38 https://www.globes.co.il/news/article.aspx?did=1001343464

39 https://www.phr.org.il/wp-content/uploads/2020/10/CovidReport_Heb_digital-the-real-final-version-compressed.pdf, p. 25–36.

40 https://www.haaretz.co.il/news/education/.premium-1.9261655

41 https://www.phr.org.il/wp-content/uploads/2020/10/CovidReport_Heb_digital-the-real-final-version-compressed.pdf, p. 38–50.

42 https://www.haaretz.co.il/news/education/.premium-1.9261655

43 https://www.btl.gov.il/Publications/oni_report/Documents/oni2018.pdf, P. 5.
